# Supplementary material for: Alteration of Metabolic Profiles during the Progression of Alzheimer’s Disease
Source: Brain Sci. 2023 Oct 13;13(10):1459. doi: 10.3390/brainsci13101459 (PMC10605479; doi:10.3390/brainsci13101459)
Supplement: Supplementary file 1 [file brainsci-13-01459-s001.zip › Supplementary Figure S1.pdf]

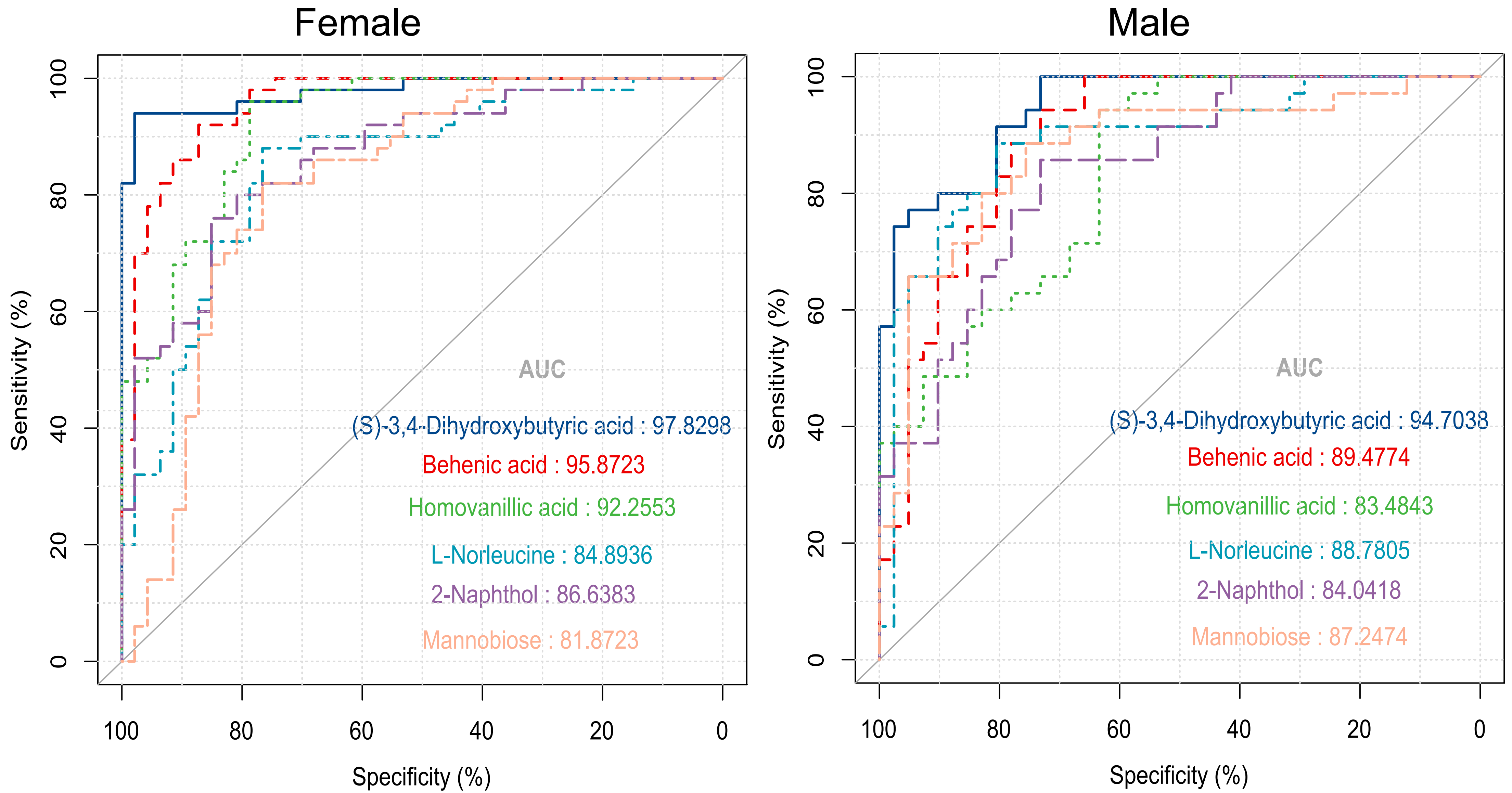

Supplementary Figure S1. The ROC curves of the six potential biomarkers in the female and male samples.
