# Supplementary material for: Alteration of Metabolic Profiles during the Progression of Alzheimer’s Disease
Source: Brain Sci. 2023 Oct 13;13(10):1459. doi: 10.3390/brainsci13101459 (PMC10605479; doi:10.3390/brainsci13101459)
Supplement: Supplementary file 1 [file brainsci-13-01459-s001.zip › Supplementary information.pdf]

Supplementary Table S1.The differentially expressed metabolites between control and AD patients.

Metabolites	Class	T-statistic	p.value	FDR
L-Norleucine	Amino acids	-10.017	7.10E-19	2.68E-17
L-Threonine	Amino acids	-8.2539	3.98E-14	6.42E-13
L-Serine	Amino acids	-7.8635	4.02E-13	4.13E-12
L-Valine	Amino acids	-7.0968	3.25E-11	2.30E-10
Pyroglutamic acid	Amino acids	-5.4507	1.73E-07	9.77E-07
L-Alanine	Amino acids	-5.2622	4.22E-07	1.83E-06
Butyric acid	Amino acids	-4.7089	5.13E-06	1.93E-05
L-Proline	Amino acids	-4.3103	2.75E-05	9.70E-05
L-alpha-Aminobutyric acid	Amino acids	-4.164	4.95E-05	0.000165
L-Cystine	Amino acids	-4.0379	8.13E-05	0.000255
L-Pipecolic acid	Amino acids	-3.8174	0.0001883	0.000507
L-Tyrosine	Amino acids	-3.3968	0.0008478	0.002083
D-Cysteine	Amino acids	-3.3265	0.0010764	0.002534
Glycine	Amino acids	-3.1231	0.0021024	0.004658
L-Glutamine	Amino acids	-3.0969	0.0022864	0.004969
L-Asparagine	Amino acids	-2.8888	0.0043681	0.008814
L-Phenylalanine	Amino acids	-2.805	0.0056147	0.010939
Creatinine	Amino acids	-2.5461	0.011777	0.021816
4-Deoxyerythronic acid	Carbohydrates and carbohydrate conjugates	-8.3269	2.57E-14	4.84E-13
Threonic acid	Carbohydrates and carbohydrate conjugates	-8.1195	8.87E-14	1.25E-12
Mannobiose	Carbohydrates and carbohydrate conjugates	8.0209	1.59E-13	2.00E-12
Sorbitol	Carbohydrates and carbohydrate conjugates	-4.9892	1.48E-06	5.77E-06
D-Glucose	Carbohydrates and carbohydrate conjugates	3.8375	0.0001747	0.000481
D-Galactose	Carbohydrates and carbohydrate conjugates	3.6038	0.000411	0.00108
Hebevinoside I	Carbohydrates and carbohydrate conjugates	3.4352	0.0007431	0.001866
Sucrose	Carbohydrates and carbohydrate conjugates	3.2546	0.0013689	0.003094
D-Arabitol	Carbohydrates and carbohydrate conjugates	-3.0737	0.0024615	0.005161
Glycerol	Carbohydrates and carbohydrate conjugates	-2.9439	0.0036919	0.007585
4-Deoxythreonic acid	Carbohydrates and carbohydrate conjugates	-2.6315	0.009277	0.017768
Erythritol	Carbohydrates and carbohydrate conjugates	-3.9586	0.0001104	0.000328
Palmitoleic acid	Lipids and lipid-like molecules	2.2968	0.022841	0.040968
Behenic acid	Lipids and lipid-like molecules	10.402	6.06E-20	3.42E-18
MG(16:0/0:0/0:0)	Lipids and lipid-like molecules	-6.4015	1.43E-09	9.50E-09
MG(0:0/18:0/0:0)	Lipids and lipid-like molecules	-3.9874	9.88E-05	0.000302
MG(18:0/0:0/0:0)	Lipids and lipid-like molecules	3.8413	0.0001722	0.000481
MG(0:0/16:0/0:0)	Lipids and lipid-like molecules	3.0731	0.0024661	0.005161
Methyl stearate	Lipids and lipid-like molecules	5.0173	1.30E-06	5.27E-06
(S)-3,4-Dihydroxybutyric acid	Organic compounds	-17.454	7.82E-40	8.84E-38
2-Naphthol	Organic compounds	8.4929	9.44E-15	2.13E-13
L-Isoleucine	Organic compounds	-7.9127	3.01E-13	3.41E-12
Homovanillic acid	Organic compounds	7.4052	5.71E-12	4.96E-11

Taurine	Organic compounds	-7.157	2.32E-11	1.75E-10
Urea	Organic compounds	-5.8347	2.64E-08	1.66E-07
Methoxamine	Organic compounds	5.625	7.44E-08	4.42E-07
Trimethoprim	Organic compounds	5.3676	2.57E-07	1.38E-06
Hexaethylene glycol	Organic compounds	-5.3437	2.88E-07	1.48E-06
Octaethylene glycol	Organic compounds	-5.3127	3.33E-07	1.64E-06
3-Methyl-2-oxovaleric acid	Organic compounds	-5.3037	3.47E-07	1.64E-06
L-Kynurenine	Organic compounds	5.1786	6.23E-07	2.61E-06
Citric acid	Organic compounds	4.5703	9.30E-06	3.39E-05
Mesylate	Organic compounds	-4.276	3.16E-05	0.000108
L-Tryptophan	Organic compounds	-3.9433	0.0001171	0.000339
Glycolic acid	Organic compounds	3.4723	0.0006536	0.001679
Heptaethylene glycol	Organic compounds	-2.8134	0.0054767	0.010857
Heneicosane	Organic compounds	-2.6136	0.0097573	0.018376
1,2,3-Trihydroxybenzene	Organic compounds	-2.3895	0.017959	0.032732
Erythrono-1,4-lactone	Organic compounds	-5.2872	3.75E-07	1.70E-06
Oxalic acid	Organic compounds	-4.0509	7.73E-05	0.000249
Hypoxanthine	Organic compounds	-3.2862	0.0012321	0.002842
Valeric acid	Sulfated fatty acids	-7.1672	2.20E-11	1.75E-10
Arachidonic acid	Unsaturated fatty acids	8.8609	1.00E-15	2.82E-14
Erucic acid	Unsaturated fatty acids	3.3891	0.0008704	0.002093

Figure legends

Supplementary figure S1. The ROC curves of the six potential biomarkers in the female and male samples.
